# Effects of family history and sex on diabetes-related outcome in type 2 diabetes – Analysis from the tyrolean diabetes registry

**DOI:** 10.1371/journal.pone.0324696

**Published:** 2025-06-18

**Authors:** Clemens Plattner, Bernhard Pfeifer, Harald Sourij, David Vill, Marietta Wiedl, Klaus Middeldorf, Egon Eisendle, Robert Eiter, Christian Ciardi, Karin Pölzl, Julia Schock, Di Chen-König, Martin Juchum, Gerald Bode, Bernhard Heindl, Christian Hengl, Karl Kirchmeyr, Lisa Rieger, Ursula Köllensberger, Andrea Schwaiger, Günther Ladner, Monika Lechleitner, Sabrina Neururer, Herbert Tilg, Susanne Kaser

**Affiliations:** 1 Department of Internal Medicine I, Gastroenterology, Hepatology & Endocrinology, Medical University Innsbruck, Innsbruck, Austria; 2 Department of Clinical Epidemiology, Tirol Kliniken Innsbruck, Innsbruck, Austria; 3 UMIT Tirol – Private University For Health Sciences and Health Technology, Hall, Austria; 4 Division of Endocrinology and Diabetology, Department of Internal Medicine, Medical University of Graz, Graz, Austria; 5 Department of Internal Medicine, Academic Teaching Hospital Hall, Hall/Tirol, Austria; 6 Department of Internal Medicine, Kufstein County Hospital, Kufstein, Austria; 7 Department of Internal Medicine, Hospital Reutte, Reutte, Austria; 8 Department of Internal Medicine, Hospital Lienz, Lienz, Austria; 9 Department of Internal Medicine, Hospital Schwaz, Schwaz, Austria; 10 Department of Internal Medicine, Hospital Zams, Zams, Austria; 11 Department of Internal Medicine, Hospital Hochzirl-Natters, Natters, Austria; 12 Department of Internal Medicine, Hospital Sankt Johann in Tyrol, St. Johann in Tyrol, Austria; 13 Department of Internal Medicine, Hospital Hochzirl-Natters, Hochzirl, Austria; 14 Rehabilitation Center Muenster, Muenster, Austria; 15 Private Internal Specialist, Woergl, Austria; 16 Private Internal Specialist, Kitzbuehel, Austria; 17 Private Internal Specialist, Schwaz, Austria; 18 Private Internal Specialist, Innsbruck, Austria; 19 Private Internal Specialist, Imst, Austria; Universita Politecnica delle Marche, ITALY

## Abstract

**Aims:**

Family history for diabetes (FHD) is a strong risk factor for type 2 diabetes (T2D), however, little is known on its effects on the outcome. Here we aimed to analyse the effects of FHD on diabetes-related outcome.

**Methods:**

7866 patients with T2D from the Tyrolean Diabetes Registry were grouped according to their FHD status. Propensity score matching for sex, BMI, HbA1c and diabetes duration provided 1440 patients per group. Survival curves were estimated using the Kaplan-Meier plot and compared using the Log-rank test.

**Results:**

Mean age at T2D diagnosis was significantly lower in the FHD group, while time to insulin initiation was independent from FHD status. FHD was associated with increased risk for neuropathy (HR 1.41 [95%CI 1.11–1.81]) but decreased risk for macrovascular disease (HR 0.84 [95%CI 0.71–0.99]). Risk for total macrovascular disease, myocardial infarction, coronary artery bypass surgery and peripheral artery disease was increased by 73–156% in males in matched groups.

**Conclusion:**

Family history for diabetes is not only associated with earlier diagnosis of type 2 diabetes but also affects diabetes-related outcomes with males being more prone to cardiovascular disease and patients with FHD to increased risk for neuropathy but decreased risk for macrovascular disease.

## Introduction

According to World Health Reports diabetes mellitus is among the 10 most common causes of death worldwide [1]. Diseases such as ischemic heart disease, stroke and kidney disease, which are well known as long-term complications of diabetes, also rank among the top ten leading causes of death worldwide stressing the importance of diabetes as driver of all-cause mortality.

Both, genetic and environmental factors contribute to the development of type 2 diabetes (T2D). Based on family and twin studies, the heritability of T2D is estimated to be as high as 25–72% [2–4]. More than 250 genomic regions have been identified to be associated with increased diabetes risk [2,5–8], however, those variants only explain a small proportion of heritability either due to rare occurrence or only modest effect [9]. Remarkably, most variants including TCF7L2 are rather linked to insulin secretion and β cell function than to insulin sensitivity. Despite the modest effects of genetic factors, family history of T2D (FHD) is associated with a nearly 3-fold increased incidence of T2D. When both parents are affected, the incidence of T2D even increases by more than 5 fold [10] suggesting that besides shared environmental and behavioural factors, genetic factors considerably contribute to type 2 diabetes risk.

Importantly, the clinical presentation and course of T2D is strongly heterogenous and diabetes-related complications are not solely predicted by glycemic control or other classical risk factors [6,11]. Only recently, phenotypic and genetic clustering have identified severe insulin resistance as major driver of cardiovascular and renal disease, while insulin deficiency was associated with increased risk for neuropathy [11–17].

The effect of sex on the course of disease is even less clear. When compared with subjects without diabetes, relative risk for cardiovascular disease was reported to be higher in middle-aged or older females than in male counterparts in some but not all studies [18–20]. Differences in risk profile and treatment intensity were discussed to explain sex-differences in diabetes-related cardiovascular outcome [21–23].

In this work we aimed to define sex-specific effects of FHD on the course of disease and diabetes-related outcomes.

## Methods

### Study cohort

We conducted an analysis of patients with T2D from a local diabetes registry (“Diabetesregister Tirol”) in Austria. Patients with type 2 diabetes who presented to hospital outpatient clinics or registered specialists between 2012 and 2020 were screened for inclusion. Clinical and laboratory data from the latest possible appointment with all information available were used for analysis. Patients with any lacking information were excluded from the study.

Registry patient records were used to determine antidiabetic medication, comorbidities, weight, HbA1c, systolic and diastolic blood pressure. Sex, body height, smoking status, time of T2D diagnosis and FHD were assessed at the initial presentation, all other clinical and laboratory assessments including BMI, HbA1c and blood pressure were obtained from the last available regular check-up. Information on smoking behaviour at time of the last available check-up was not available why smoking behaviour was not included in analysis.

A positive FHD was defined as at least one first degree family member with known T2D (i.e., parents or siblings). Insulin free survival was defined as the time from the diagnosis of diabetes according to registry data until the start of insulin therapy.

Microvascular disease was defined by presence of diabetic kidney disease, retinopathy and/or neuropathy. Diabetic kidney disease was defined as sustained albuminuria (urinary albumin/creatinine ratio >30 mg/g) or estimated glomerular filtration rate (eGFR) reduction below 60 ml/min/1.73m². Macrovascular disease includes myocardial infarction and/or stroke, established coronary artery disease including history of aortocoronary bypass surgery or percutaneous coronary intervention and/or diagnosis of or intervention for peripheral artery disease, respectively.

The local ethics committee of the Medical University of Innsbruck stated that at the time of submission no ethics committee approval was required for retrospective observational studies/ case reports by Austrian law. Accordingly, no consent was obtained from study participants. All relevant data are within the manuscript and its Supporting information files.

### Statistical analysis

Descriptive data analysis was performed for all parameters and data were shown as means ± standard deviation (SD). Patients were grouped according to their FHD status. Continuous parameters were summarized by means and SD at last available check-up. Propensity score matching was performed for BMI (last visit), HbA1c (last visit), sex and diabetes duration (last visit) without consideration of other cardiovascular confounders.

Chi-squared analysis was used to test the association between categorical variables. A student’s t-test was performed to compare the means of two independent groups. The p-value was considered significant if p < 0.05.

Event-free survival curves for insulin initiation, microvascular and macrovascular disease were produced using the Kaplan-Meier method and were compared by log-rank test in propensity score matched groups. Kaplan-Meier plots were created using duration of diabetes and insulin initiation, micro- and macrovascular complications as events and log rank tests were used to compare FHD vs non-FHD groups or sexes in matched groups.

## Results

### Baseline characteristics

In total, 11,838 patients with T2D who presented to the participating health providers from 2012 to 2020 were screened for inclusion. 7,866 patients with all clinical and laboratory data available were included in the study. 3,635 had a positive family history for type 2 diabetes (FHD group) while 4,231 patients did not have a family history of type 2 diabetes (non-FHD group).

Clinical characteristics of all included patients are shown in Table 1. At study inclusion FHD patients were younger and had a longer diabetes duration. Mean BMI and HbA1c levels were higher in the FHD group and also the female/male ratio was higher in the FHD group.

**Table 1:** Clinical characteristics.

	FHD	Non-FHD	p-value
total number	3635	4231	
age (years)	64.3 ± 12.57	67.55 ± 11.60	<0.01
female (%)	45.28	37.46	<0.01
BMI (kg/m²)	29.91 ± 6.04	29.54 ± 5.71	<0.01
HbA1c (mmol/mol) [%]	57 ± 15	57 ± 15	0.03
HbA1c (%)	7.4 ± 1.4	7.3 ± 1.4	
systolic blood pressure (mmHg)	133 ± 29	133 ± 44	0.89
diastolic blood pressure (mmHg)	79 ± 17	79 ± 17	0.67
diabetes duration (years)	11.39 ± 8.70	13.03 ± 9.76	<0.01
duration of insulin treatment (years)	6.41 ± 2.56	6.45 ± 2.57	0.71
microvascular complications (%)	17.80	18.84	0.40
macrovascular complications (%)	17.03	19.97	<0.01
diabetic kidney disease (%)	11.42	14.18	<0.01
retinopathy (%)	2.94	1.77	<0.01
neuropathy (%)	8.42	6.64	<0.01
myocardial infarction (%)	8.89	8.79	0.76
stroke (%)	4.24	5.58	0.01
peripheral artery disease (%)	3.82	5.11	0.34
coronary bypass (%)	9.07	9.88	0.35

Clinical characteristics of all included patients are shown. Total number of patients is given or mean values ± standard deviation unless otherwise specified. BMI denominates as body mass index, FHD as family history for diabetes, non-FHD as patients without family history for diabetes and HbA1c as haemoglobin A1c. Sex, body height, time of T2D diagnosis and FHD were assessed at the initial presentation, all other clinical and laboratory assessments were obtained from the last available regular check-up.

Age at diabetes diagnosis was significantly lower in the FHD group when compared with the non-FHD group (51.89 + /-12.13 vs. 56.59 + /-11.80, p < 0.01). Differences in age at diagnosis remained significant when female and male patients were analysed separately (females: FHD group 52.82 + /-12.73 vs 57.18 + /-12.16 years in the non-FHD group; p < 0.01; males: FHD group 51.12 + /-11.57 vs 56.24 + /-11.56 years in the non-FHD group; p < 0.01) (Supplementary S1 Table).

### Time to event analysis in propensity score matched groups

In order to define the effect of FHD on macrovascular and microvascular outcome propensity score analyses were performed in a 1:1 match. Groups were matched for sex, BMI, HbA1c and diabetes duration. Characteristics of the groups are shown in Table 2 and Table 3.

**Table 2:** Characteristics of propensity score matched groups.

	FHD	Non-FHD	p-value
total number	1440	1440	
age (years)	66.79 ± 11.10	66.66 ± 10.70	0.75
female (%)	39.51	40.70	0.54
BMI (kg/m²)	29.61 ± 5.79	29.78 ± 5.74	0.41
HbA1c (mmol/mol) [%]	58 ± 14	58 ± 15	0.85
HbA1c (%)	7.4 ± 1.3	7.4 ± 1.4	
systolic blood pressure (mmHg)	136 ± 23	135 ± 23	0.42
diastolic blood pressure (mmHg)	80 ± 13	80 ± 12	0.14
diabetes duration (years)	7.8 ± 7.19	7.70 ± 6.73	0.70

Characteristics of propensity score matched groups are shown. Groups were matched in a propensity score model for diabetes duration, BMI, HbA1c and sex. Total number of patients is given or mean values ± standard deviation unless otherwise specified. BMI denominates as body mass index, FHD as family history for diabetes, non-FHD as patients without family history for diabetes and HbA1c as haemoglobin A1c.

**Table 3:** Characteristics of propensity score matched groups.

	FHD females	Non-FHD females	p-value FHD vs Non-FHD	FHD males	Non-FHD males	p-value FHD vs non -HD
total number	569	586		871	854	
age (years)	69.72 ± 10.7	68.67 ± 10.3	0.09	64.87 ± 10.86	65.28 ± 10.69	0.43
BMI (kg/m²)	30.02 ± 6.57	30.34 ± 6.27	0.39	29.34 ± 5.24	29.40 ± 5.31	0.81
HbA1c (mmol/mol)	59 ± 15	59 ± 15	0.78	57 ± 14	57 ± 15	0.70
HbA1c (%)	7.6 ± 1.4	7.5 ± 1.4		7.4 ± 1.3	7.4 ± 1.4	
systolic blood pressure (mmHg)	136 ± 25	135 ± 22	0.65	135 ± 21	134 ± 23	0.49
diastolic blood pressure (mmHg)	80 ± 14	80 ± 12	0.56	81 ± 12	80 ± 13	0.15
diabetes duration (years)	7.90 ± 7.11	7.70 ± 6.37	0.62	7.73 ± 7.25	7.70 ± 6.97	0.92

Characteristics of propensity score matched groups separately for females and males are shown. Groups were matched in a propensity score model for diabetes duration, BMI, HbA1c and sex. Total number of patients is given or mean values ± standard deviation unless otherwise specified. BMI denominates as body mass index, FHD as family history for diabetes, non-FHD as patients without family history for diabetes and HbA1c as haemoglobin A1c.

Antidiabetic medication was similar in patients with and without FHD (S2 Table).

**Fig 1:**
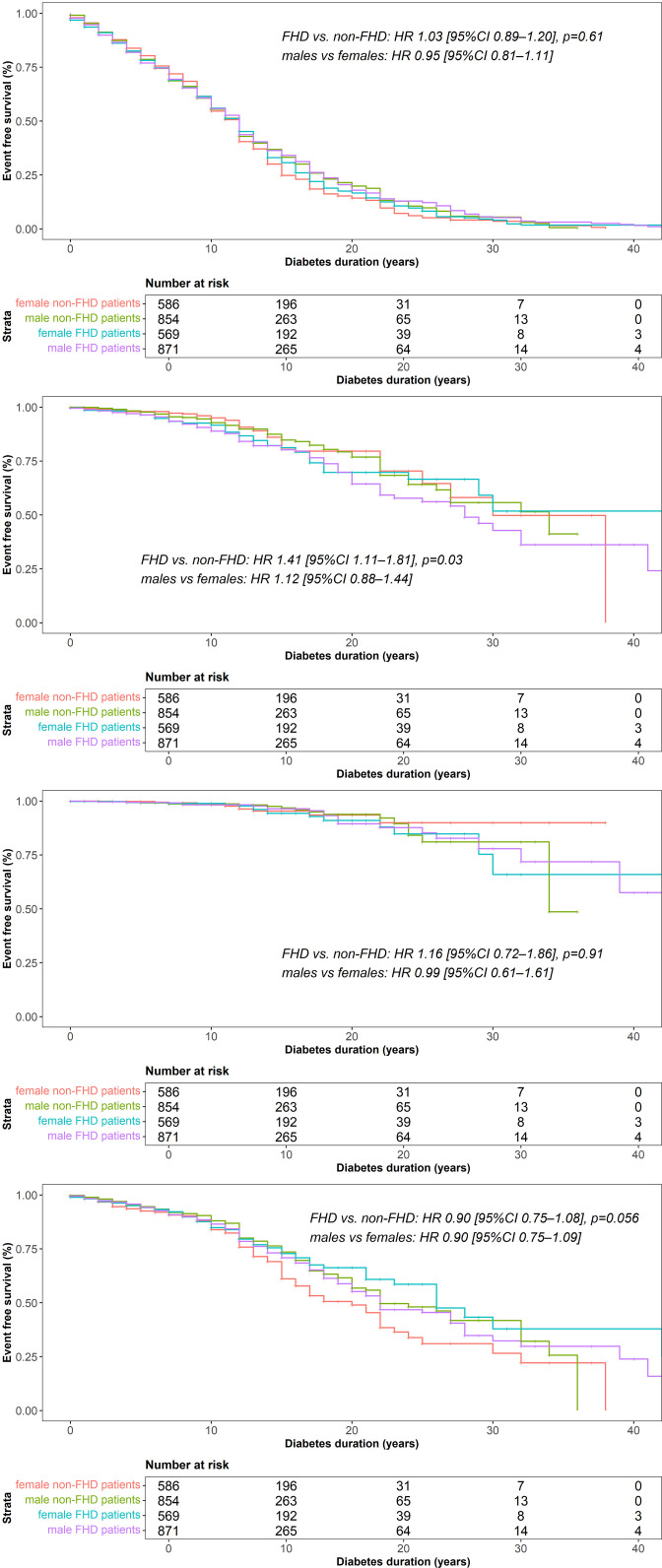
Event-free survival analysis of microvascular outcome. Kaplan Meier curves for event free from a) microvascular disease in total, b) neuropathy, c) retinopathy and d) diabetic kidney disease are shown. Log-rank test was performed to compare between FHD and non FHD groups and males and females in propensity score matched groups for diabetes duration, sex, HbA1c and BMI and hazard ratios (HR) with confidence intervals (CI) are shown. *Red line, female non-FHD patients, green line, male non-FHD patients, blue line: female FHD patients, purple line, male FHD patients.*

Insulin-free survival was similar in FHD and non-FHD patients (S1 Fig). Kaplan Meier plots for microvascular disease are shown in Fig 1.

Neither FHD nor sex significantly influenced event-free survival for a composite of incident microvascular disease including neuropathy, retinopathy and diabetic kidney disease (Fig 1a) (FHD vs. non-FHD: (HR 1.03 [95%CI 0.89–1.20], males vs females: (HR 0.95 [95%CI 0.81–1.11]). Remarkably, risk for neuropathy was significantly higher in the FHD group (HR 1.41 [95%CI 1.11–1.81], p = 0.03) (Fig 1b) while no differences were found for retinopathy and diabetic kidney disease between the FHD and non-FHD group (Figs 1c,d) (retinopathy: (HR 1.15 [95%CI 0.72–1.86], diabetic kidney disease: (HR 0.90 [95%CI 0.75–1.08]). Event-free survival for neuropathy, retinopathy or diabetic kidney disease was not affected by sex in matched groups (Figs 1b-d) (neuropathy: (HR 1.12 [95%CI 0.88–1.44], retinopathy: (HR 0.99 [95%CI 0.61–1.61], diabetic kidney disease: (HR 0.90 [95%CI 0.75–1.09]).

**Fig 2:**
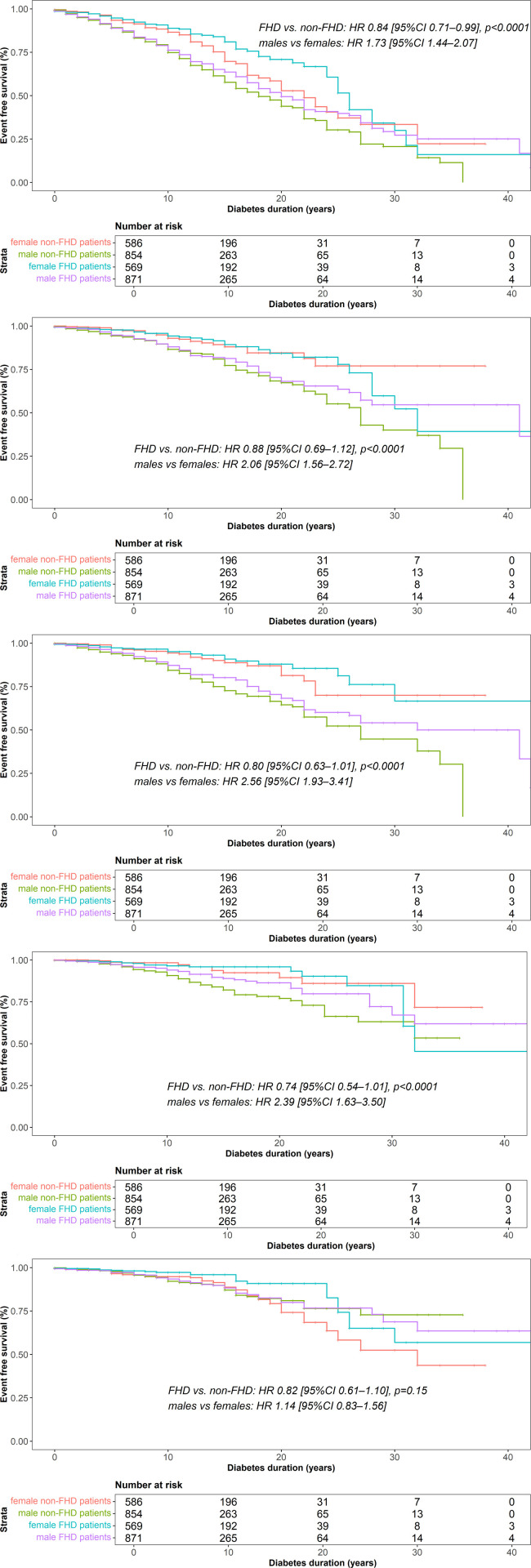
Event-free survival analysis of macrovascular outcome. Kaplan Meier curves for event free from a) macrovascular disease in total, b) myocardial infarction c) coronary artery bypass surgery, d) peripheral artery disease and e) stroke are shown. Log-rank test was performed to compare between FHD and non FHD groups and males and females in propensity score matched groups for diabetes duration, sex, HbA1c and BMI and hazard ratios (HR) with confidence intervals (CI) are shown. *Red line, female non-FHD patients, green line, male non-FHD patients, blue line: female FHD patients, purple line, male FHD patients.*

When risk for macrovascular disease encompassing acute myocardial infarction, stroke, peripheral artery disease and coronary artery bypass surgery was analysed, Kaplan Meier plot showed decreased risk in the FHD group (HR 0.84 [95%CI 0.71–0.99], p < 0.01) and increased risk in males (HR 1.73 [95%CI 1.44–2.07], p < 0.01) for the composite of macrovascular disease, respectively (Fig 2a). When macrovascular endpoints were analysed separately, risk was higher in males for acute myocardial infarction (HR 2.07 [95%CI 1.56–2.73], p < 0.01) (Fig 2b), coronary artery bypass surgery (HR 2.56 [95%CI 1.93–3.41], p < 0.01) (Fig 2c), peripheral artery disease (HR 2.39 [95%CI 1.64–3.50], p < 0.01) (Fig 2d), respectively, while no difference was found between the FHD and the non-FHD group (acute myocardial infarction (HR 0.88 [95%CI 0.70–1.12], coronary artery bypass surgery: (HR 0.80 [95%CI 0.63–1.01], peripheral artery disease (HR 0.74 [95%CI 0.54–1.01]. Stroke risk was comparable between the FHD and non-FHD group and females and males, respectively (FHD vs non-FHD: (HR 0.82 [95%CI 0.61–1.10], males vs. females: (HR 1.14 [95%CI 0.83–1.56] (Fig 2e).

## Discussion

A positive FHD is an independent and well-established risk factor for T2D [10] and accordingly is explicitly taken into account in international screening recommendations for T2D [24]. While risk for T2D in offspring has extensively been investigated only little is known how positive FHD affects the course of disease and the risk for diabetes-associated complications.

By analysing data of the Tyrolean Diabetes Registry we aimed to more precisely define the role of FHD on the course of disease. The Tyrolean Diabetes Registry Database covers patients who present to metabolic outpatient clinics of public hospitals in Tyrol or selected registered specialists in internal medicine. In this study, data were analysed from patients who have been continuously treated for at least 5 years in participating outpatient clinics or by registered doctors until the beginning of 2020. Data from 2020 have not been used for analysis as influence of SARS-CoV2 pandemic on diabetes management and diagnosis of diabetes-associated diseases cannot fully be ruled out.

In our study T2D was diagnosed at an earlier age in both female and male patients with FHD. Our findings are in line with several previous studies also suggesting that positive FHD is associated not only with increased risk but also earlier diagnosis of T2D [25–27].

Causally, diminished beta cell function [28,29] and insulin action in offspring of patients with T2D have been proposed to explain increased diabetes risk [29,30]. Accordingly, genome-wide association studies have shown that candidate genes in T2D are rather associated with beta cell development or insulin secretion than with insulin resistance [31]. Besides environmental factors, earlier or more efficient screening in offspring of patients with T2D might also explain earlier diagnosis in these patients. Remarkably, insulin-free survival was independent from family history status in our study suggesting that earlier initiation of insulin treatment in patients with FHD might rather reflect earlier age at diagnosis than faster or more advanced decline of beta cell function.

When analysing the risk for diabetes-related comorbidities in propensity score matched groups, we found that patients with FHD display increased neuropathy risk, while no significant effects on retinopathy and nephropathy was found. In a recent study defining novel subgroups of diabetes in a 5 year follow up study, prevalence of diabetic sensorimotor polyneuropathy was highest in patients with severe insulin-deficient diabetes [15]. Importantly, beta cell function is impaired even in non-obese offspring of patients with type 2 diabetes [32,33], suggesting that glucose homeostasis might be impaired due to an early decline in beta cell function in persons with FHD a long time before being diagnosed with type 2 diabetes. Accordingly, increased glucose excursions have been reported in offspring of patients with T2D [34]. Importantly, a high glycemic variability which is usually found in patients with insulin deficiency has been shown to be a risk factor for diabetic neuropathy independent of HbA1c and diabetes duration [35]. Thus, we hypothesize that early impairment of beta cell function in offspring of T2D patients and increased glucose excursions might explain increased risk of neuropathy in diabetic patients with FHD.

In contrast to neuropathy, pathophysiology of retinopathy and diabetic kidney disease is not limited to glucose toxicity but also associated with hypertension [36] or other cardiovascular risk factors [37] which as a consequence might attenuate the effect of FHD on microvascular disease other than neuropathy.

Very strikingly, in our study macrovascular risk was significantly higher in patients without FHD. Besides advanced age, male sex, overweight or obesity and dysglycemia, blood pressure, dyslipidemia and smoking are other well-established risk factors for cardiovascular disease. In our study, blood pressure levels were comparable in patients with and without FHD. As a limitation of our study, LDL-cholesterol levels and data on current smoking behaviour were not available in this registry. However, all patients were treated according to national guidelines irrespective of their family history status making it unlikely, that differences in LDL-cholesterol might explain increased macrovascular risk in patients without FHD. Besides these classical risk factors, insulin resistance turned out to be a strong predictor of cardiovascular risk: Recent cluster analysis identified a subgroup of patients with severe insulin resistance who displayed highest cardiovascular and renal risk while patients with severe insulin deficiency displayed increased risk for diabetic neuropathy [11,15]. Underlining these phenotypic subgroups, genetic characterization of the original clusters from the ANDIS cohort revealed that the severe insulin-resistant diabetes cluster was associated with the polygenic score for fasting insulin reflecting insulin resistance but was not associated with any polygenic score for insulin deficiency [12,38,39]. When applying results from these subgroup analyses to our study [11,15] non-FHD patients might clinically most likely resemble the cluster of severe insulin resistance with increased cardiovascular risk.

We thus suggest that insulin resistance might play an even more striking role in development of T2D and diabetes-related comorbidities in patients without FHD than in those with FHD. These data might suggest that insulin resistance should especially be addressed in T2D treatment in patients without FHD while patients with FHD might clinically most likely resemble the cluster of insulin deficiency with increased neuropathy risk [11,15].

Interestingly, cardiovascular morbidity and mortality was found increased in non-diabetic offspring of patients with type 2 diabetes [40,41]. Furthermore, a family history for type 2 diabetes increased the risk for nephropathy and cardiovascular disease in patients with type 1 diabetes [42,43] suggesting that offspring of patients with type 2 diabetes display higher cardiorenal risk. Interestingly, in our study, risk of cardiovascular disease was higher in patients without FHD than in those with FHD. This apparent discrepancy might be explained by the surpassing detrimental role of insulin resistance on cardiovascular disease in patients without FHD.

Very remarkably, total macrovascular risk as well as risks for myocardial infarction, coronary artery bypass surgery and peripheral artery disease were significantly higher in male than in female matched groups. Previous landmark studies showed that while cardiovascular typically occurs 10 years earlier in males, this gap does not exist in patients with type 2 diabetes underlining the importance of dysglycemia and insulin resistance on atherogenesis. Nevertheless, and in accordance with our data, absolute risk of cardiovascular mortality is higher in males than in female patients with type 2 diabetes [18,19,22,23]. Mechanistically, higher underlying cardiovascular risk in men and inaccuracy of BMI estimating visceral obesity might explain greater cardiovascular risk in male T2D patients in our study.

In our study, sex-specific differences might not be due to differences in glycemic control or BMI. Due to lacking data differences in smoking behaviour and LDL-cholesterol cannot be fully ruled out to partly explain the difference, however, lipid lowering treatment was performed according to international guidelines in a sex-independent way which make it very unlikely that LDL-C levels were significantly different between males and females in our study.

In conclusion our data suggest that male patients with type 2 diabetes are at especially high risk for cardiovascular risk.

In summary, our analysis demonstrated increased risk of neuropathy in patients with FHD, while patients without FHD are more prone to macrovascular disease. Irrespective of their FHD status, male patients displayed higher risk for cardiovascular morbidity.

From our data we suggest that patients without FHD might clinically rather predispose to the phenotypic subgroup of severe insulin resistance while those with FHD rather predispose clinically to the phenotypic cluster of insulin deficiency. However, this hypothesis will need to be further explored in datasets containing the required parameters to do subgroup clustering. Taking together, status of family history and sex should be taken into account in risk stratification and management of T2D.

## Supporting information

S1 Table Clinical characteristics of female and male study participants. Total number of patients is given or mean values + /- standard deviation unless otherwise specified. BMI denominates as body mass index, FHD as family history for diabetes, non-FHD as patients without family history for diabetes and HbA1c as hemoglobin A1c.
(DOCX)

S2 Table Antidiabetic medication in propensity score matched groups. Groups were matched in a propensity score model for diabetes duration, BMI, HbA1c and sex. Data are shown as percentage of patients. FHD as family history for diabetes, non-FHD as patients without family history for diabetes, GLP-1 as glucagon like peptide-1, SGLT-2 as sodium glucose transporter-2.
(DOCX)

S1 Fig Insulin-free survival in patients with and without FHD. Kaplan Meier curves for insulin free survival are shown. Log-rank test was performed to compare between FHD and non FHD groups and males and females in propensity score matched groups for diabetes duration, sex, HbA1c and BMI and hazard ratios (HR) with confidence intervals (CI) are shown. *Red line, female non-FHD patients, green line, male non-FHD patients, blue line: female FHD patients, purple line, male FHD patients.*
(TIFF)
