# Supplementary material for: Effects of family history and sex on diabetes-related outcome in type 2 diabetes – Analysis from the tyrolean diabetes registry
Source: PLoS One. 2025 Jun 18;20(6):e0324696. doi: 10.1371/journal.pone.0324696 (PMC12176189; doi:10.1371/journal.pone.0324696)
Supplement: S1 Table — Total number of patients is given or mean values + /- standard deviation unless otherwise specified. BMI denominates as body mass index, FHD as family history for diabetes, non-FHD as patients without family history for diabetes and HbA1c as hemoglobin A1c. (DOCX) [file pone.0324696.s001.docx]

|  | FHD  females | Non-FHD  females | p-value  FHD vs non-FHD females | FHD  males | Non-FHD  males | p-value  FHD vs non-FHD males |
| --- | --- | --- | --- | --- | --- | --- |
| total number | 1646 | 1585 |  | 1989 | 2646 |  |
| age (years) | 65.57±12.88 | 68.71±11.80 | <0.01 | 61.94±12.20 | 66.86±11.40 | <0.01 |
| BMI (kg/m²) | 30.66±6.89 | 29.97±6.28 | <0.01 | 29.32±5.16 | 29.28±5.31 | 0.85 |
| HbA1c (mmol/mol) | 58 ±14.65 | 57±15.19 | 0.78 | 57±14.75 | 56 ±14.75 | 0.09 |
| HbA1c (%) | 7.5±1.3 | 7.4±1.4 |  | 7.4±1.4 | 7.3 ± 1.4 |  |
| systolic blood pressure (mmHg) | 133±30 | 134±29 | 0.64 | 133±28 | 133±29 | 0.86 |
| diastolic blood pressure (mmHg) | 79±18 | 79±16 | 0.67 | 79±16 | 79±17 | 0.85 |
| diabetes duration (years) | 11.78±8.89 | 13.53±9.84 | <0.01 | 11.07±8.68 | 12.73±9.68 | <0.01 |
| duration of insulin treatment (years) | 4.52±2.67 | 4.45±2.63 | 0.62 | 7.97±2.72 | 7.65±2.62 | <0.01 |
| microvascular complications (%) | 17.74 | 18.61 | 0.70 | 17.85 | 18.97 | 0.48 |
| macrovascular complications (%) | 10.75 | 13.82 | 0.02 | 22.22 | 23.66 | 0.39 |
| nephropathy (%) | 11.00 | 14.95 | <0.01 | 11.76 | 13.72 | 0.08 |
| retinopathy (%) | 3.10 | 1.89 | 0.03 | 2.82 | 1.70 | 0.01 |
| neuropathy (%) | 8.00 | 5.49 | <0.01 | 8.60 | 6.95 | 0.03 |
| myocardial infarction (%) | 5.71 | 5.36 | 0.64 | 11.51 | 10.85 | 0.40 |
| stroke (%) | 3.40 | 5.24 | 0.02 | 4.93 | 5.78 | 0.27 |
| peripheral artery disease (%) | 2.37 | 2,65 | 0.75 | 5.03 | 6.58 | 0.04 |
| coronary artery bypass (%) | 4.80 | 5.43 | 0.53 | 12.62 | 12.55 | 0.83 |
