# Supplementary material for: Effects of family history and sex on diabetes-related outcome in type 2 diabetes – Analysis from the tyrolean diabetes registry
Source: PLoS One. 2025 Jun 18;20(6):e0324696. doi: 10.1371/journal.pone.0324696 (PMC12176189; doi:10.1371/journal.pone.0324696)
Supplement: S2 Table — Groups were matched in a propensity score model for diabetes duration, BMI, HbA1c and sex. Data are shown as percentage of patients. FHD as family history for diabetes, non-FHD as patients without family history for diabetes, GLP-1 as glucagon like peptide-1, SGLT-2 as sodium glucose transporter-2. (DOCX) [file pone.0324696.s002.docx]

| Antidiabetic medications (%) | FHD | Non-FHD | p-value |
| --- | --- | --- | --- |
| metformin | 60.3 | 58.0 | 0.23 |
| SGLT-2 inhibitors | 20.1 | 21.9 | 0.27 |
| GLP-1 agonists | 8.8 | 9.5 | 0.52 |
| gliptin | 31.5 | 32.8 | 0.50 |
| sulfonylurea | 6.2 | 6.3 | 0.94 |
| ⍺-glucosidase inhibitors | 0.3 | 0.1 | 0.37 |
| glitazone | 5.5 | 5.1 | 0.68 |
| insulin | 53.7 | 53.1 | 0.77 |
