# Supplementary figures and images for: Effects of family history and sex on diabetes-related outcome in type 2 diabetes – Analysis from the tyrolean diabetes registry
Source: PLoS One. 2025 Jun 18;20(6):e0324696. doi: 10.1371/journal.pone.0324696 (PMC12176189; doi:10.1371/journal.pone.0324696)

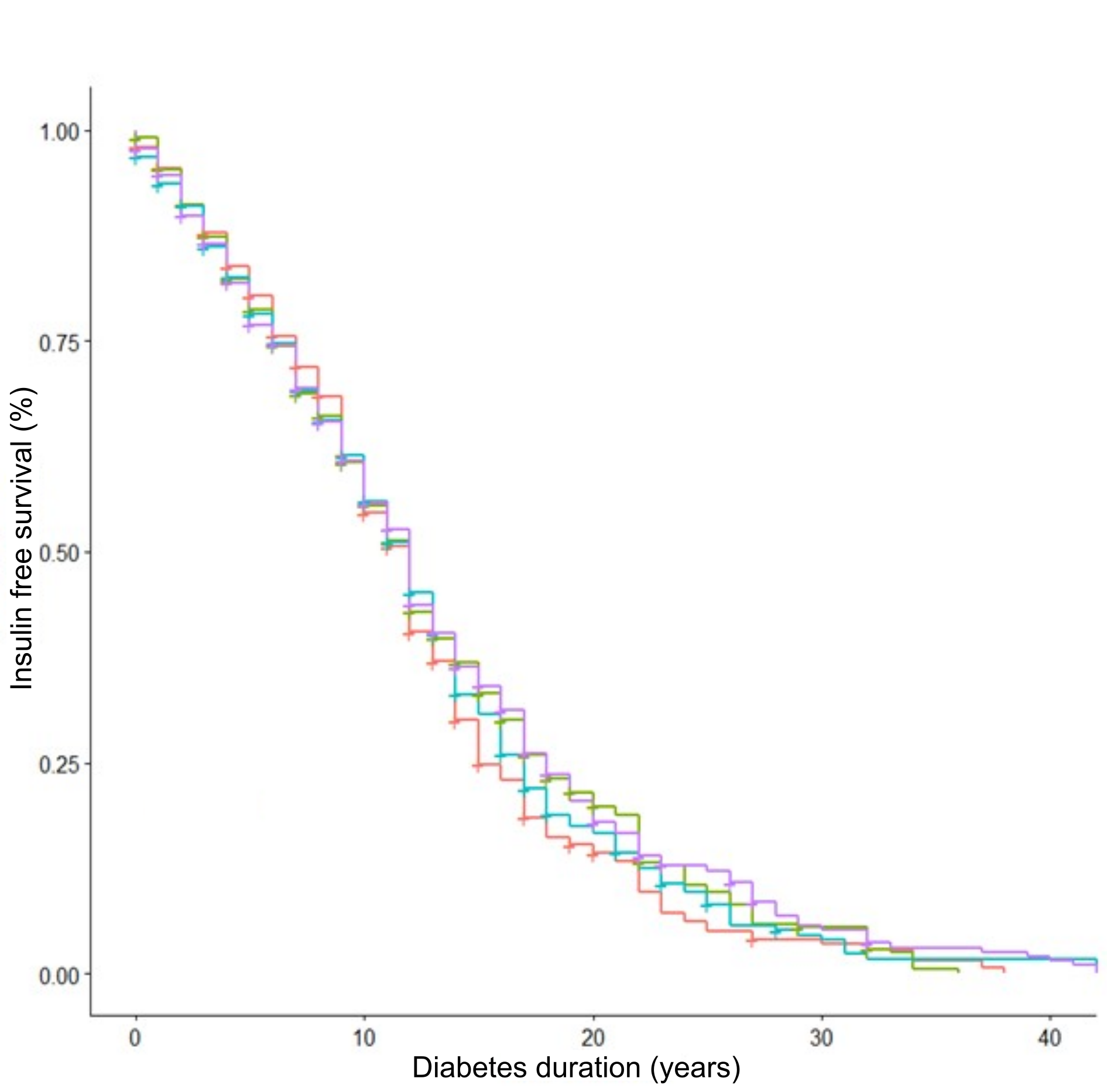

Supplement: S1 Fig — Kaplan Meier curves for insulin free survival are shown. Log-rank test was performed to compare between FHD and non FHD groups and males and females in propensity score matched groups for diabetes duration, sex, HbA1c and BMI and hazard ratios (HR) with confidence intervals (CI) are shown. Red line, female non-FHD patients, green line, male non-FHD patients, blue line: female FHD patients, purple line, male FHD patients. (TIFF) [file pone.0324696.s003.tiff]
